# Evidence for the multiple hits genetic theory for inherited language impairment: a case study

**DOI:** 10.3389/fgene.2015.00272

**Published:** 2015-08-24

**Authors:** Tracy M. Centanni, Jordan R. Green, Jenya Iuzzini-Seigel, Christopher W. Bartlett, Tiffany P. Hogan

**Affiliations:** ^1^Massachusetts General Hospital Institute of Health Professions, Boston, MAUSA; ^2^Massachusetts Institute of Technology, Cambridge, MAUSA; ^3^Marquette University, Milwaukee, WIUSA; ^4^The Ohio State University, Columbus, OHUSA

**Keywords:** specific language impairment, multiple hit model, gene association, language disorders, 15q11.2

## Abstract

Communication disorders have complex genetic origins, with constellations of relevant gene markers that vary across individuals. Some genetic variants are present in healthy individuals as well as those affected by developmental disorders. Growing evidence suggests that some variants may increase susceptibility to these disorders in the presence of other pathogenic gene mutations. In the current study, we describe eight children with specific language impairment and four of these children had a copy number variant in one of these potential susceptibility regions on chromosome 15. Three of these four children also had variants in other genes previously associated with language impairment. Our data support the theory that 15q11.2 is a susceptibility region for developmental disorders, specifically language impairment.

## Introduction

Specific language impairment (SLI) is a developmental language disorder characterized by impaired oral language skills (Leonard et al., 1999; Catts et al., 2005). The disorder is typically diagnosed in the preschool years, when children normally begin speaking in more complex and complete sentences. These children have normal non-verbal IQ in spite of their problems with semantics, syntax, and discourse (Paul, 2007). Hallmark grammatical errors include the omission of articles (such as “the”), pronoun mistakes (e.g., “him” in place of “he”), grammatical inflection (e.g., “go” instead of “goes”), and tense errors (e.g., switching present for past tense).

Children with language impairments rarely have a single gene mutation and it is agreed that even individuals with the same disorder are unlikely to have the exact same set of genetic markers (Bishop, 2002). The lack of a consistent causal gene has led some to speculate that complex developmental disorders such as dyslexia, attention deficits, and SLI are instead due to any one of several combinations of genetic markers. Recently, evidence has suggested that some genetic variants may create a susceptibility to developmental disorders, and these susceptibility variants may be common in many individuals, even if the remainder of their genetic variants differ (Donlon, 1988; Wang et al., 2009; Burnside et al., 2011; Centanni et al., 2015; Hashemi et al., 2015).

The effect of copy number variants (CNVs) on chromosome 15 (q11.2) has been a subject of debate in the field, both anecdotally and in scholarly articles. Microdeletions and microduplications in this region have been associated with a variety of disorders, including autism, schizophrenia, Prader–Willi, and Angelman’s syndromes (Kirov et al., 2009; Hogart et al., 2010; Mefford et al., 2010; Dimitropoulos et al., 2013; Hashemi et al., 2015). However, duplications at this location are commonly seen in typically developing individuals (Mefford et al., 2010), which raises questions about whether variants at this location play a role in the disorders mentioned above. The consistent association between this region and a variety of developmental disorders suggests that variants in this region do contribute to the disordered state, even if they are not causal on their own. Though copy number variations in this region have been suggested as a susceptibility variant in many disorders, it is currently unknown if these variants are susceptibility factors for disorders such as SLI.

In the current report, we discuss the behavioral and genetic profiles of eight children with SLI who took part in a larger study on the biological basis of language impairment. Due to the current controversy regarding the definition of SLI and its diagnostic criterion (Reilly et al., 2014), we used strict assessment score cutoffs that are in line with other studies on the genetics of SLI (Rice et al., 2009). Four of these children all had gains in this region of chromosome 15 as well as additional CNVs in multiple other regions previously linked to language impairments.

## Materials and Methods

### Participants

In the current study, we discuss four children who were part of a cohort of eight children with SLI, ranging in age from 4;5 to 17;2 (years;months), that participated in a larger study on the biological pathways of speech and language disorders. All procedures were approved by the Institutional Review Board of the University of Nebraska Medical Center and all participants were consented prior to participation. Participants completed a series of commonly administered, age-appropriate speech, language, reading, and cognitive assessments including the *Goldman Fristoe Test of Articulation-Second Edition* (*GFTA-2;* Goldman and Fristoe, 2000), the *Clinical Evaluation of Language Fundamentals-Fourth Edition* (*CELF-4;* Semel et al., 2003), *Reynolds Intellectual Assessment Scales* (*RIAS;* Reynolds and Kamphaus, 2005), and the *Woodcock Reading Mastery Test-Revised* (*WRMT-R;* Woodcock, 1998). All participants were required to have normal cognition based on a standard score higher of 75 or higher on the *RIAS.*

Children were assigned to the SLI group based on *GFTA-2* percentile scores of 16 or higher and a *CELF-4* standard score below 85. Inclusionary criteria are presented in **Table 1**.

**Table 1:** Inclusionary criterion for categorization in the specific language impairment (SLI) group.

	Non-verbal IQ^1^	Speech production^2^	Language^3^	Word reading^4^
Criterion	>75	>16th percentile	<85	Any

*^1^Reynolds Intellectual Assessment Scales (RIAS; Reynolds and Kamphaus, 2005).*

*^2^Goldman-Fristoe Test of Articulation (GFTA-2; Goldman and Fristoe, 2000).*

*^3^Clinical Evaluation of Language Fundamentals (CELF-4; Semel et al., 2003).*

*^4^Woodcock Reading Mastery Test (WRMT-R; Woodcock, 1998).*

### DNA Collection and Isolation

Buccal cell samples were collected from all eight participants with SLI using the Isohelix DNA swab packs (Cell Projects, Ltd., Kent, UK), and DNA was extracted per manufacturer’s recommendations using the QIACube (Qiagen, Valencia, CA, USA). DNA quantity and quality were determined using the NanoDrop ND-1000^®^ spectrophotometer (NanoDrop Technologies, Wilmington, DE, USA) and agarose gel electrophoresis, respectively.

### CNV Detection

High-resolution genome-wide analysis was performed on genomic DNA using the CytoScanHD^TM^ array (Affymetrix, Santa Clara, CA, USA) according to manufacturer’s instruction. This array contains more than 2.6 million markers for high-resolution whole-genome copy number analysis and 750,000 genotype-able single nucleotide polymorphisms (SNPs) for reliable detection of copy neutral loss of heterozygosity (CN-LOH). Data were visualized and analyzed with the Chromosome Analysis Suite (ChAS) software (Affymetrix) using the following filter parameters: (1) ≥25 markers and ≥5 kilobases (kb) for CNVs and (2) ≥5 megabases (Mb) for CN-LOH. All basepairs are mapped to Build 37/hg19. Parental DNA samples were not available for these children, so it was not possible to determine whether these were *de novo* variants.

### Statistical Analysis

We used Pearson’s correlation to evaluate the relationship between gain size in 15q11.2 and phenotype characteristics (*p* < 0.05).

## Results

### Behavioral Profile

All eight children were administered a number of speech, language, and cognitive assessments to ensure a diagnosis of SLI in the absence of any comorbid conditions (**Table 1**). All children were classified as having SLI since they scored below 85 on the language measure in the presence of normal non-verbal IQ and no articulation impairments (**Table 2**). Because children five through eight did not evidence any variants at 15q11.2, their data were excluded from further consideration in this paper. Child 1 was 10;3 (years;months) and female, child 2 was 9;3 and female, child 3 was 10;1 and female, and child 4 was 11;3 and male. None of the children scored within the impaired range on the word reading measure (<85), but they did display a wide range of typical word reading abilities, from the 30th percentile (child 1, 30; child 2, 32; child 3, 45) up to the 73rd percentile (child 4).

**Table 2:** Assessment standard scores (percentiles in parenthesis where applicable) for four children with copy number variants (CNVs) at 15q11.2.

	Age (months)	Non-verbal IQ^1^	Speech production^2^	Language^3^	Word reading^4^
Child 1	124	103 (58)	101 (36)	70 (2)	92 (30)
Child 2	112	104 (61)	103 (42)	64 (1)	93 (32)
Child 3	121	98 (45)	100 (36)	64 (1)	98 (45)
Child 4	136	101 (53)	105 (34)	82 (12)	109 (73)

*Age is displayed in months.*

*^1^Reynolds Intellectual Assessment Scales (RIAS; Reynolds and Kamphaus, 2005).*

*^2^Goldman-Fristoe Test of Articulation (GFTA-2; Goldman and Fristoe, 2000).*

*^3^Clinical Evaluation of Language Fundamentals (CELF-4; Semel et al., 2003).*

*^4^Woodcock Reading Mastery Test (WRMT-R; Woodcock, 1998).*

### Genetic Profile

Of the eight children with SLI that were genotyped, four of these children had large gains in an overlapping region at 15q11.2. Child 1’s gain was 54.34 kilo-bases in length (25283093–25337431), child 2 had a gain of 41.70 kb (25295728–25337431), child 3 had a gain of 32.94 kb (25291742–25324677), and child 4 had a gain of 14.81 kb (25306864–25321675). These gains are large and encompass a variety of genes. Because of the size of these gains, exons and introns for a variety of genes were affected. Most hits among these four children included the genes SNORD109A, SNORD109B, and SNORD116-(1-23). These genes are commonly associated with Prader–Willi syndrome, with evidence suggesting that several genes, including SNORD116, are a key pathogenic component (Rabinovitz et al., 2012; Anderlid et al., 2014).

We also annotated copy number at other known language, and more broadly, neurodevelopmental loci. Overall, the range of assessment scores and the variety of gain sizes in the sample suggest that other genetic factors may be contributing to the observed phenotypes (**Table 3**). Child 1 had two additional significant gains or losses (hereafter collectively called ‘hits’). The first was a gain at 13q21.1, which has been seen in individuals with autism and language impairment (Bartlett et al., 2004). The second was a loss at 12p13.33, which has been previously associated with childhood apraxia of speech (CAS) and attention difficulties (Thevenon et al., 2013). Child 2 had two hits of clinical significance in addition to 15q11.2. The first was a loss at 10q21.1, which has been associated with intellectual disability, lack of expressive speech, and attention deficit and hyperactivity disorder (ADHD; Neale et al., 2010; Freunscht et al., 2013). The second was a loss at 16p11.2, which has been previously associated with autism (Kumar et al., 2008; Weiss et al., 2008; Laffin et al., 2012). This gene is often characterized as pathogenic and likely contributed to the phenotype of this child.

**Table 3:** Additional CNVs for each of four children with hits at 15q11.2.

	CNV Identified	Type	Size (kbp)	Linear location	Previous associations	Prevalence in Database of Genomic Variants (DGV)	Prevalence in Ontario Population Genomics Platform (OPGP)
Child 1	13q21.1	Gain	76.78	57713219–57789996	Autism and language impairment (Bartlett et al., 2004).	3,267 out of 26,404 subjects (12.37%)	213 out of 873 subjects (24.51%)
	12p13.33	Loss	24.01	2235940–2259954	CAS and attention(Thevenon et al., 2013)	514 out of 27,543 subjects (1.86%)	89 out of 873 subjects (10.2%)
Child 2	10q21.1	Loss	30.84	53383971–53414806	Intellectual disability, lack of speech and attention deficit and hyperactivity disorder (ADHD; Neale et al., 2010; Freunscht et al., 2013)	8 out of 22,208 subjects (0.003%)	2 out of 873 subjects (0.02%)
	16p11.2	Loss	579.42	29597822–30177240	Autism, schizophrenia(Kumar et al., 2008; Weiss et al., 2008; Laffin et al., 2012)	317 out of 22,680 subjects (1.39%)	Not found
Child 3	9p24.3	Loss	17	291610–308949	Intellectual disability (de Vries et al., 2005; Griggs et al., 2008)	94 out of 23,637 subjects (0.39%)	Not found
	22q13.33	Gain	103.33	51072547–51175872	SHANK3 is an autism gene, duplication noted with developmental delay (Durand et al., 2007; Moessner et al., 2007)	129 out of 22,634 subjects (0.05%)	48 out of 873 subjects (5.49%)
Child 4	7q11.23	Gain	38.21	74162950–74201156	Expressive language delay and the Williams–Beuren locus (Jurado et al., 1998; Somerville et al., 2005; Berg et al., 2007)	4 out of 45 subjects (8.8%)	Not found

Child 3 had two CNVs in addition to the deletion at 15q11.2, both of which may be pathogenic. The first was a loss at 9p24.3 involving the gene *DOCK8* and has been previously linked with intellectual disability (de Vries et al., 2005; Griggs et al., 2008). In fact, this child did have the lowest non-verbal IQ (standard score of 98) of the four children described here (**Table 2**). The second was a gain at 22q13.33. A duplication of this region is linked with developmental delay and the region also contains the gene *SHANK3*, which has been associated with autism (Durand et al., 2007; Moessner et al., 2007). Finally, child 4 had one CNV in addition to the deletion at 15q11.2: a gain at 7q11.23, which has been associated with language delay and the Williams–Beuren locus (Jurado et al., 1998; Somerville et al., 2005; Berg et al., 2007). The result that these children all exhibited other genetic variants previously associated with speech and language impairments support the idea that a variant at 15q11.2 is a susceptibility locus and not necessarily one that is deleterious by itself.

**FIGURE 1:**
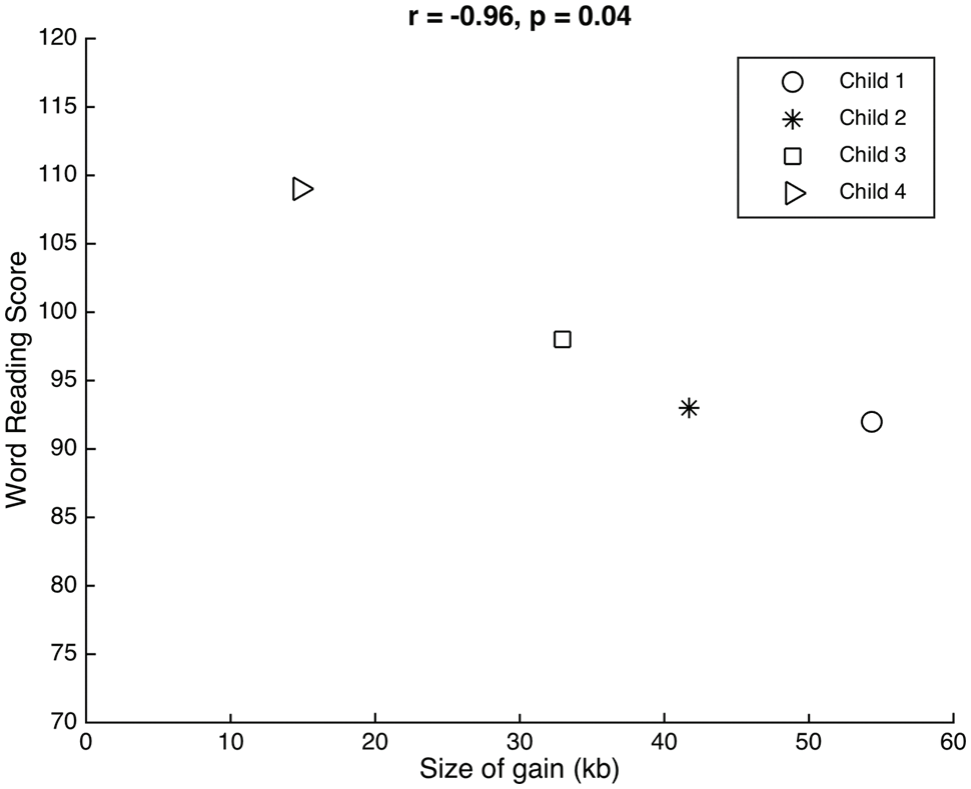
**Relation between gain size and word reading.** There was a significant correlation between the size of the gain observed at 15q11.2 in each of the children and their respective scores on the word reading measure.

The genetic profiles have an interesting phenotypic context for evaluating the function of 15q11.2 relative to reading and language. We consider the clustering of percentiles for children 1–3 and the relative outlier of child 4. Child 4 had the smallest gain (18.13 kb less than the next largest gain, in child 3) and also had the highest scores on the language and word reading measures. Although this child is the oldest in our sample, it is unlikely that age was a factor considering that the reading scores were normed for age. Although there are just four data points, there is a significant linear association between the size of the gain observed and the scores on the word reading measure (*r* = -0.96, *p* = 0.04; **Figure 1**). In spite of the age correction, Child 4’s data point could be a possible outlier. Future studies with a larger group of children are required to validate this potential association between gain size and word reading scores.

### Prevalence of Observed Hits in the General Population

Search of the Database of Genomic Variants (DGV; http://dgv.tcag.ca/) yielded one unselected sample with variation at 15q11.2. A single deletion was found (1 out of *N* = 873 subjects) in the Ontario Population Genomics Platform (OPGP) controls (Costain et al., 2013). This study used the same technology as our study (Affymetrix-CytoScanHD). To evaluate the population prevalence of the secondary hits observed in our four children, we also searched the DGV and the OPGP for each of the variants reported here. Population frequencies in these two populations are shown in **Table 3**.

## Discussion

In the current study, we report the behavioral phenotypes of four children with SLI who also had large gains in the q11.2 region of chromosome 15. These children all had poor oral language abilities compared to typical peers. In spite of normal non-verbal intelligence and normal speech articulation, these children had a wide range of abilities in word reading. Three of the four children also had additional genetic variants located in areas previously associated with speech and language impairments. These results support the theory that variants at 15q11.2 may create an increased predisposition to displaying a language disorder.

### Strengths and Caveats of the Current Study

A strength of the current study is the strict criterion used to identify children with SLI. Because this disorder often co-occurs with dyslexia (Leonard et al., 2002; McCarthy et al., 2012), it has been difficult to determine which genes are related to SLI specifically and which are related to dyslexia. Though our sample size was small (eight children with SLI), the result that four of the eight had a duplication at 15q11.2 supports previous work linking developmental disorders with microdeletions or microduplications in this region (de Kovel et al., 2010; Hashemi et al., 2015). Since all the children in our study were confirmed as having SLI, we were unable to provide support for previous reports that this CNV can occur in typically developing individuals. Future studies should investigate this marker in a larger population of typically developing children as well as those with SLI in the absence of comorbid conditions.

### The Multiple Hits Model of Developmental Disorders

To date, no single genetic marker reliably predicts the occurrence of SLI. It is likely that SLI, and perhaps other communication disorders, are caused by a constellation of genes (Rice et al., 2009). An existing hypothesis states that region 15q11.2 is a susceptibility variant. If so, a hit in this region could increase the likelihood that an individual will exhibit a developmental disorder phenotype when additional risk variants are also present. Microdeletions in this region are commonly associated with developmental disorders like Prader–Willi Syndrome (Dimitropoulos et al., 2013) and Angelman Syndrome (Donlon, 1988), as well as epilepsy (Mefford et al., 2010) and autism (de Kovel et al., 2010). For example, a microdeletion in 15q11.2 was observed in 1% of individuals with idiopathic generalized epilepsies (12 of 1234; de Kovel et al., 2010). These deletions are often seen in unaffected family members in addition to affected offspring.

Though the variants seen in the current study were microduplications rather than microdeletions, recent evidence suggests that this type of variant may also indicate susceptibility to developmental disorders, including autism (Hogart et al., 2010; Kitsiou-Tzeli et al., 2010; van der Zwaag et al., 2010) and speech delay (Burnside et al., 2011). The consistent observation that microduplications in 15q11.2 are associated with SLI in our sample, together with previous evidence that microdeletions in this area are related to other developmental disorders, suggests that this region is sensitive to mutations of various forms. It is interesting to note that the four children with gains at 15q11.2 did not evidence any variants in regions previously associated with SLI, including 16q (SLI1), 19q (SLI2), and 13q (SLI3) (Bartlett et al., 2004; Consortium, 2004; Monaco, 2007; Newbury et al., 2011). Specifically, a locus at 16q known as SLI1, has not only been linked with SLI in a large sample, but is also associated with basic reading, spelling, and reading comprehension measures (Consortium, 2004). The observation that none of our participants exhibited variants in these notable regions is likely due to a combination of study design and sample size. Developmental language and communication disorders are notorious for having a complicated genetic picture, without a single causal gene (Bishop, 2002). It is possible that the variant at 15q represents another path to SLI in the absence of variants at the previously associated areas.

Our result provides additional support to 15q11.2 as a susceptibility locus, though larger studies of persons with language and related cognitive phenotypes are needed to establish the prevalence of this variant in the general population compared with a variety of developmental disorders.

## Conflict of Interest Statement

The authors declare that the research was conducted in the absence of any commercial or financial relationships that could be construed as a potential conflict of interest.
